# Endobronchial epithelioid leiomyoma (leiomyoblastoma) causing complete right-lung atelectasis: A case report

**DOI:** 10.1097/MD.0000000000050599

**Published:** 2026-09-04

**Authors:** Bassel Ibrahim, Raneem Ahmad, Youssef Abbas, Mais Salleh, Mohammad Alaa Aldakak, Youness Souleiman

**Affiliations:** a National University Hospital, Faculty of Medicine, Damascus University, Damascus, Syrian Arab Republic; b Faculty of Medicine, Damascus University, Damascus, Syrian Arab Republic.

**Keywords:** bilobectomy, complete lung atelectasis, endobronchial leiomyoblastoma, epithelioid leiomyoma, right main bronchus obstruction

## Abstract

**Rationale:**

Endobronchial epithelioid leiomyoma, historically termed leiomyoblastoma, is an exceptionally rare smooth-muscle tumor that can cause critical airway obstruction and mimic malignant endobronchial disease.

**Patient concerns:**

A 45-year-old never-smoking woman presented with several months of progressive exertional dyspnea and a persistent dry cough. Examination revealed absent breath sounds over the right hemithorax and rightward tracheal deviation.

**Diagnoses:**

Chest radiography and computed tomography demonstrated complete right-lung atelectasis with ipsilateral mediastinal shift. Bronchoscopy showed a smooth, white mass completely occluding the right main bronchus. Biopsy was deferred because an endobronchial hydatid cyst and a vascular tumor remained possible. Histopathology after resection showed epithelioid cells with clear-to-eosinophilic cytoplasm, minimal atypia, <2 mitoses/10 high-power fields, and focal coagulative necrosis. Tumor cells were diffusely positive for desmin, with focal weak cytokeratin and vimentin positivity; Ki-67 was below 5%.

**Interventions:**

Right posterolateral thoracotomy revealed a polypoid tumor arising from the right upper-lobe bronchus and extending into the right main bronchus. Because the upper lobe was extensively destroyed and the fissural anatomy precluded meaningful parenchymal preservation, right upper and middle bilobectomy was performed while preserving the lower lobe.

**Outcomes:**

Resection margins and lymph nodes were tumor-free. The right lower lobe re-expanded satisfactorily, recovery was uneventful, and the patient remained asymptomatic approximately 13 months after surgery.

**Lessons:**

Rare benign endobronchial tumors can cause complete unilateral lung collapse. Complete resection with maximal feasible parenchymal preservation is essential, and long-term surveillance is warranted because of uncertain biological behavior.

## 1. Introduction

Benign tumors of the tracheobronchial tree are uncommon, and leiomyomas account for <2% of benign lung tumors.^[1]^ These lesions are generally slow-growing and may produce nonspecific symptoms until substantial airway obstruction has developed. Reported manifestations of benign endobronchial tumors include cough, dyspnea, wheezing, hemoptysis, and recurrent post-obstructive infection. Radiological findings may include atelectasis, bronchiectasis, recurrent pneumonia, or mediastinal shift, and may therefore mimic a malignant process.^[2,3]^

Epithelioid leiomyoma, historically termed leiomyoblastoma, is a rare smooth-muscle neoplasm characterized by predominantly round or polygonal epithelioid cells with eosinophilic or clear cytoplasm.^[4,5]^ Although leiomyoblastomas and epithelioid leiomyomas have been described at several anatomical sites, involvement of the tracheobronchial tree is exceptionally uncommon.^[4]^

A structured literature search of PubMed/MEDLINE and Google Scholar was conducted from database inception through July 2026 using combinations of the terms “leiomyoblastoma,” “epithelioid leiomyoma,” “bizarre leiomyoma,” “endobronchial,” “bronchial,” “tracheobronchial,” and “airway.” Only 1 previous publication specifically reporting an endobronchial leiomyoblastoma was identified.^[6]^ To the best of our knowledge, the present case therefore represents the 2nd published report specifically designated as endobronchial leiomyoblastoma.

We report a 45-year-old woman with complete obstruction of the right main bronchus and total right-lung atelectasis caused by an endobronchial epithelioid leiomyoma (leiomyoblastoma). This case illustrates the diagnostic difficulty posed by rare benign endobronchial tumors and the need to individualize surgical management when parenchymal-sparing resection is not anatomically feasible.

## 2. Case presentation

A 45-year-old Arab woman, an office employee who had never smoked or consumed alcohol, presented with several months of progressively worsening exertional dyspnea. Her symptoms had gradually progressed to modified Medical Research Council grade III and were accompanied by a persistent dry cough. She denied chest pain, hemoptysis, fever, night sweats, weight loss, anorexia, or fatigue. Previous treatment for presumed asthma with bronchodilators and corticosteroids had provided only partial symptomatic relief. Her medical history was notable for postoperative deep-vein thrombosis following abdominal myomectomy 5 years earlier. The histopathological report of the uterine lesion was unavailable. She also reported an allergy to levofloxacin.

On examination, the patient was afebrile, with a blood pressure of 130/70 mm Hg, heart rate of 75 beats/min, respiratory rate of 24 breaths/min, and oxygen saturation of 95% on room air. Chest examination demonstrated reduced right-sided expansion, absent tactile fremitus and breath sounds over the right hemithorax, dullness to percussion, and tracheal deviation toward the right. The remainder of the physical examination was unremarkable.

**Figure 1.**
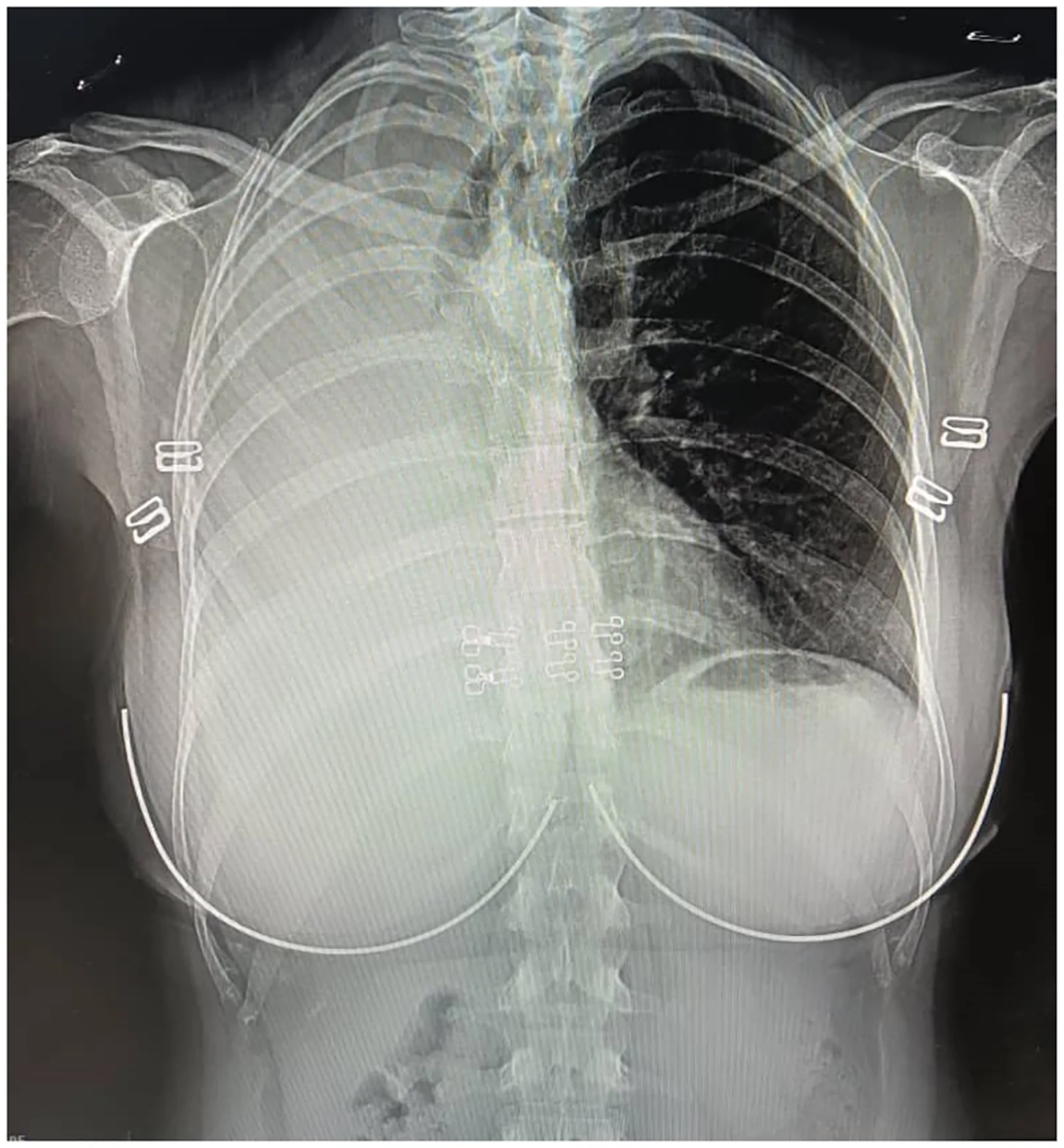
Preoperative chest radiograph. Chest radiograph demonstrating complete opacification of the right hemithorax with ipsilateral tracheal and mediastinal deviation, consistent with total right-lung atelectasis and marked volume loss.

**Figure 2.**
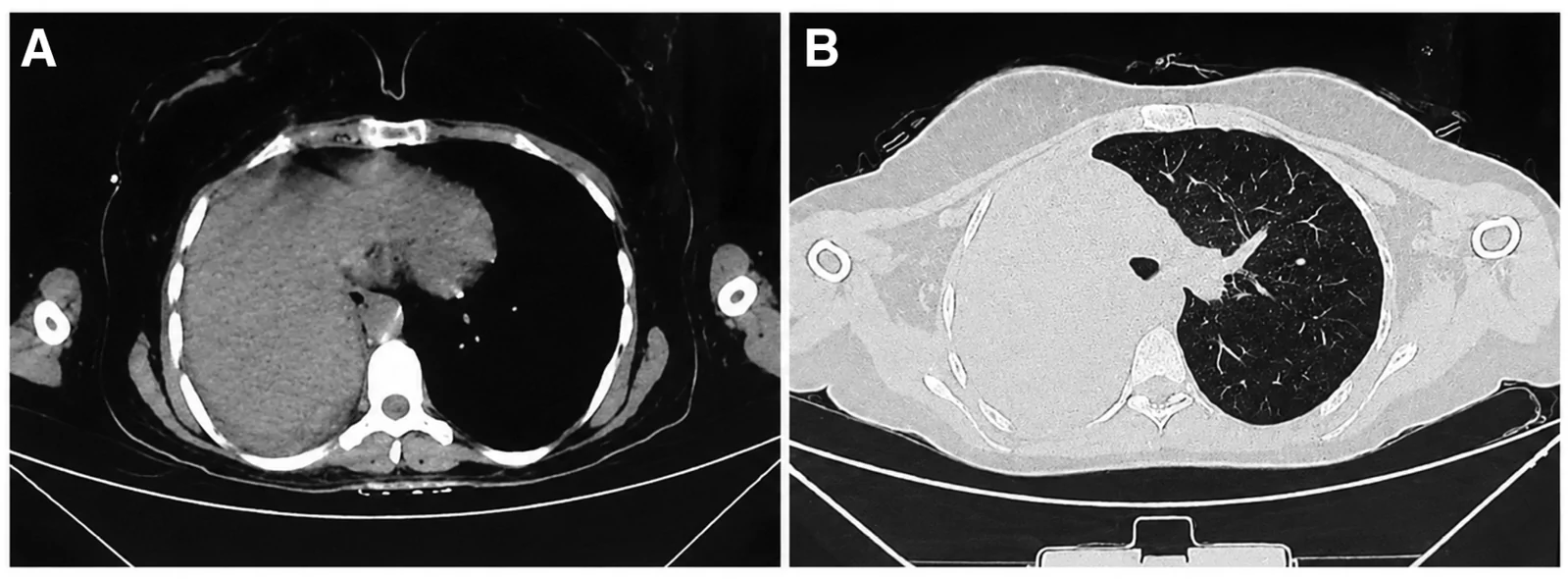
Preoperative chest computed tomography. Axial non-contrast chest CT images. (A) Mediastinal window showing total collapse of the right lung with marked rightward mediastinal shift and a small ipsilateral pleural effusion. (B) Lung window demonstrating complete right-lung atelectasis with compensatory hyperinflation of the left lung. CT = computed tomography.

During the initial evaluation, chest radiography demonstrated complete opacification of the right hemithorax with ipsilateral tracheal deviation, consistent with complete right-lung atelectasis (Fig. 1). Non-contrast chest computed tomography (CT) performed the following day confirmed total right-lung collapse with marked mediastinal shift toward the right, elevation of the right hemidiaphragm, and a minimal right pleural effusion (Fig. 2A and B).

**Figure 3.**
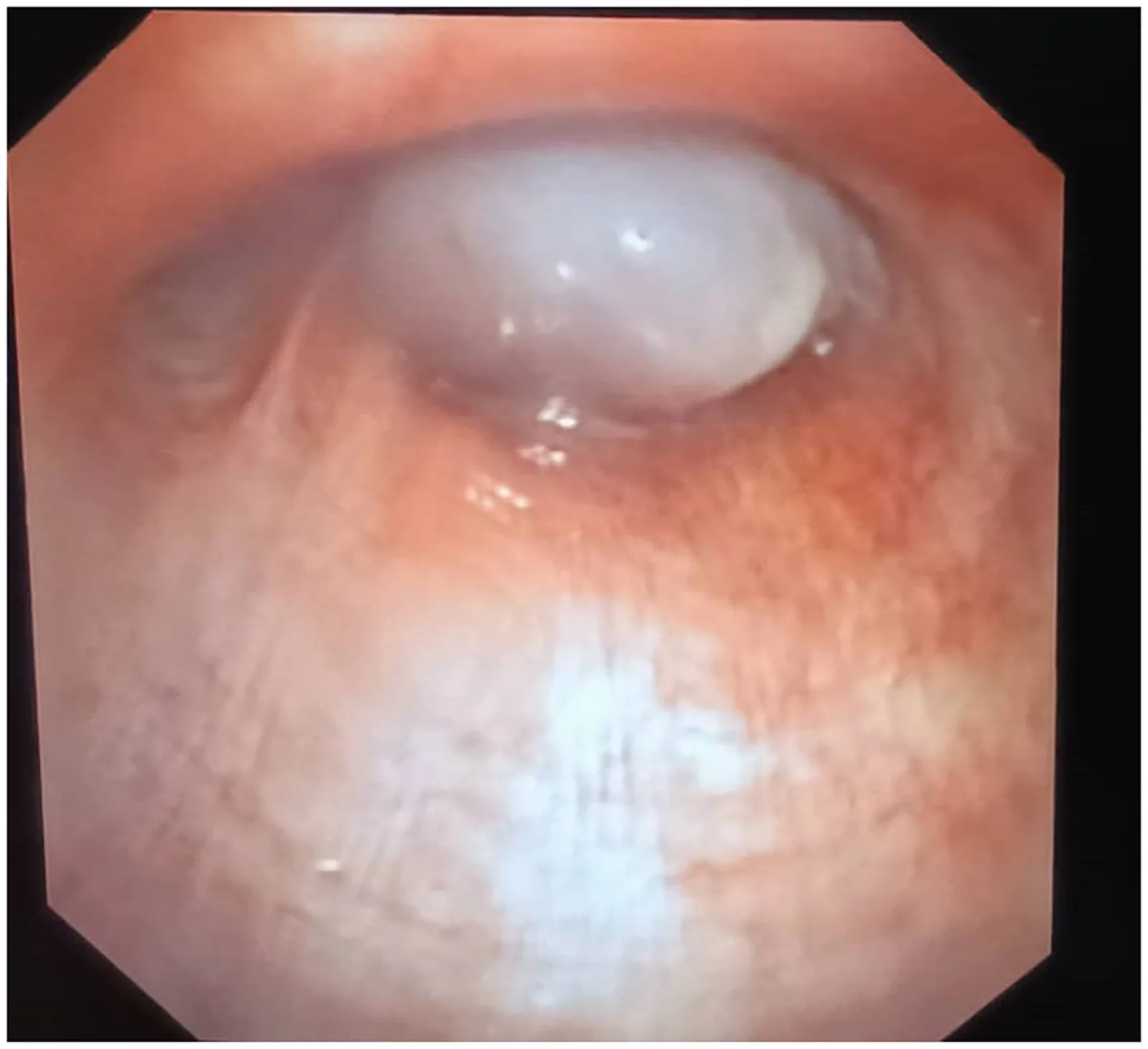
Bronchoscopic appearance of the endobronchial tumor. Flexible bronchoscopy showing a smooth, white, cyst-like endobronchial mass completely occluding the right main bronchus and preventing advancement of the bronchoscope beyond the obstruction.

Flexible bronchoscopy performed approximately 1 week later revealed a smooth, white, cyst-like endobronchial lesion completely occluding the right main bronchus (Fig. 3). The bronchoscope could not be advanced beyond the lesion. The trachea, left main bronchus, larynx, and vocal cords appeared normal. Bronchoscopic biopsy was deferred because the lesion’s smooth cystic appearance raised concern for an endobronchial hydatid cyst, in which instrumentation could have resulted in rupture, intrabronchial spillage, airway flooding, and anaphylaxis. A vascular endobronchial tumor, particularly a carcinoid tumor, was also considered, raising an additional concern regarding substantial bleeding. These potential complications were especially concerning because the right main bronchus was completely obstructed, and ventilation was functionally dependent on the contralateral lung.

Laboratory investigations demonstrated leukocytosis of 16,000/mm^3^ with 85% neutrophils. Sputum culture grew *Staphylococcus aureus* susceptible to linezolid. The patient received linezolid for 1 week according to the antimicrobial susceptibility result, with subsequent improvement in the leukocyte count. A repeat sputum culture obtained before surgery was sterile. Preoperative pulmonary function testing was not performed because marked dyspnea limited the patient’s ability to cooperate reliably with the examination. In addition, the completely atelectatic right lung was considered functionally noncontributory, and management was primarily determined by the fixed central airway obstruction and the need to restore ventilation.

Approximately 6 days after bronchoscopy, the patient underwent right posterolateral thoracotomy through the 5th intercostal space under general anesthesia. Following adhesiolysis, a polypoid tumor arising from the right upper-lobe bronchus was identified, extending into and completely obstructing the right main bronchus. The right upper lobe was extensively destroyed and largely replaced by a solid tumor.

**Figure 4.**
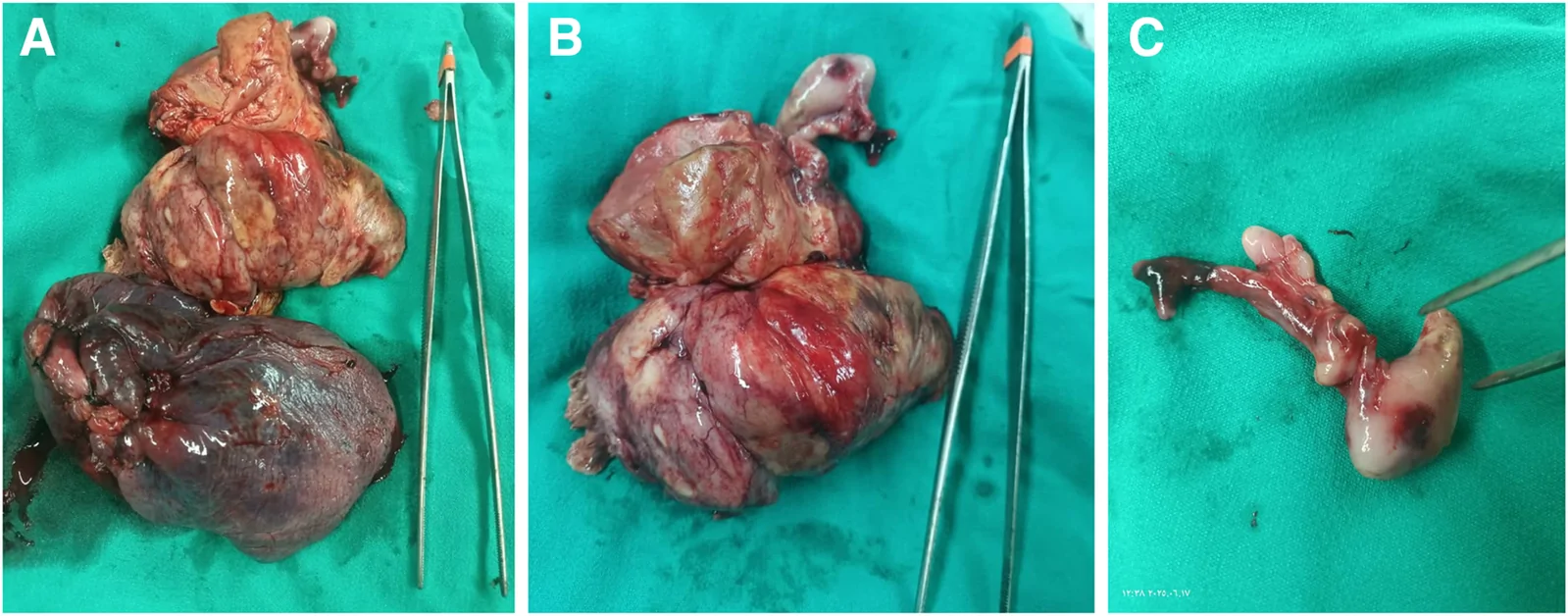
Gross appearance of the resected specimen. (A) Right upper- and middle-lobectomy specimen showing extensive replacement of the right upper lobe by a bulky solid tumor. (B) Closer view of the lobulated resected tumor and associated lung specimen. (C) Gross appearance of the polypoid endobronchial component extracted from the right upper-lobe bronchus, which had extended into and obstructed the right main bronchus.

**Figure 5.**
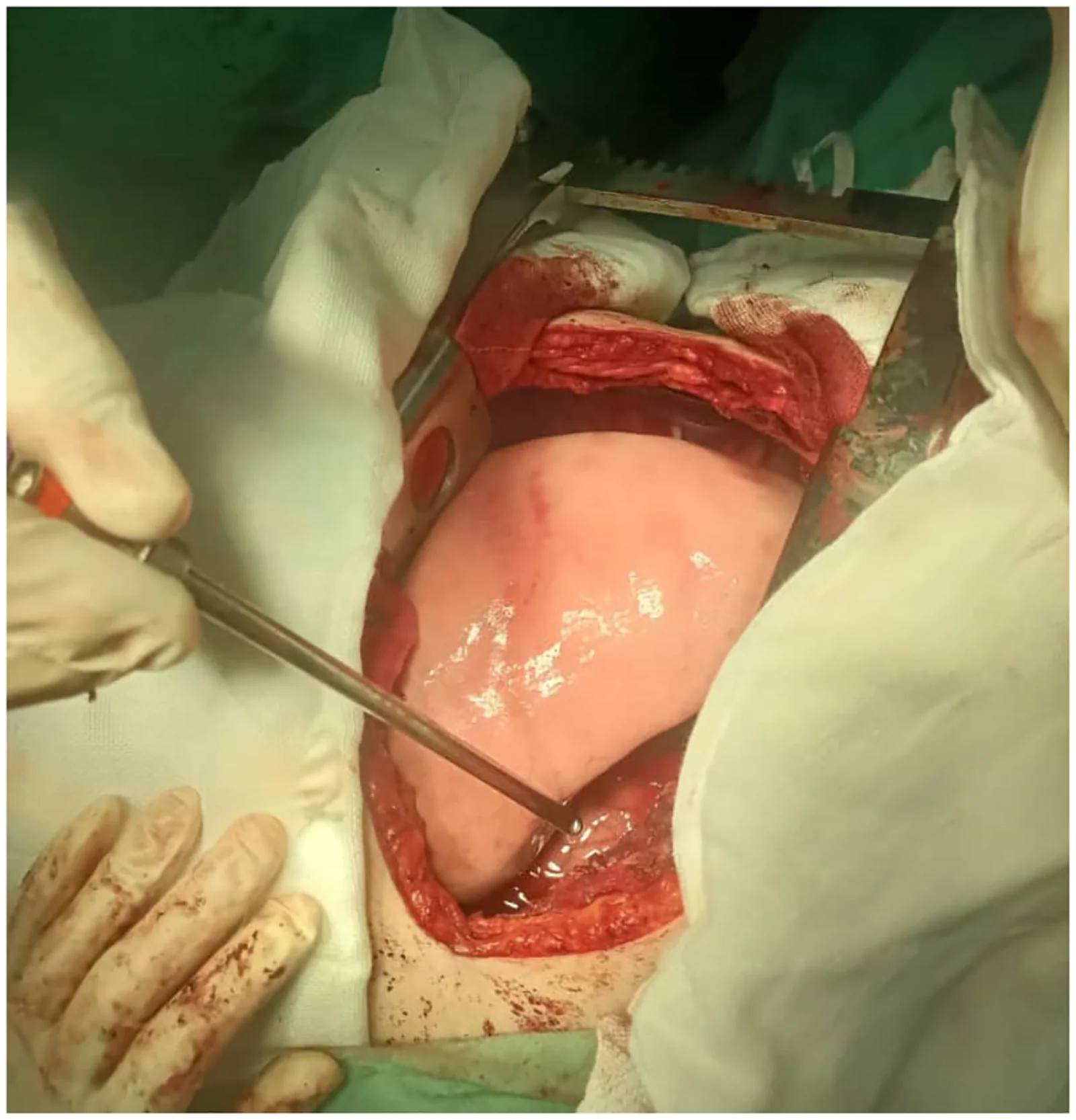
Intraoperative re-expansion of the preserved right lower lobe. Intraoperative view following right upper and middle bilobectomy and removal of the obstructing endobronchial tumor, demonstrating satisfactory re-expansion and ventilation of the preserved right lower lobe.

A sleeve or bronchoplastic resection was considered but was not anatomically appropriate because no functional upper-lobe parenchyma remained to be preserved. Furthermore, the fissure between the upper and middle lobes was incompletely developed, and the 2 lobes formed a closely integrated anatomical unit. The bronchus intermedius was not involved. A right upper and middle bilobectomy was therefore performed to achieve complete resection while preserving the unaffected right lower lobe. The upper-lobe bronchus was opened, and the endobronchial polyp was extracted from deep within the right main bronchus. The gross bilobectomy specimen and bulky tumor are shown in Figure 4A and B, while the extracted endobronchial polypoid component is shown in Figure 4C. Following tumor removal, the right lower lobe re-expanded satisfactorily with good ventilation (Fig. 5). The bronchial stumps were closed, systematic hilar and mediastinal lymph-node dissection was performed, hemostasis was secured, and a chest tube was inserted.

**Figure 6.**
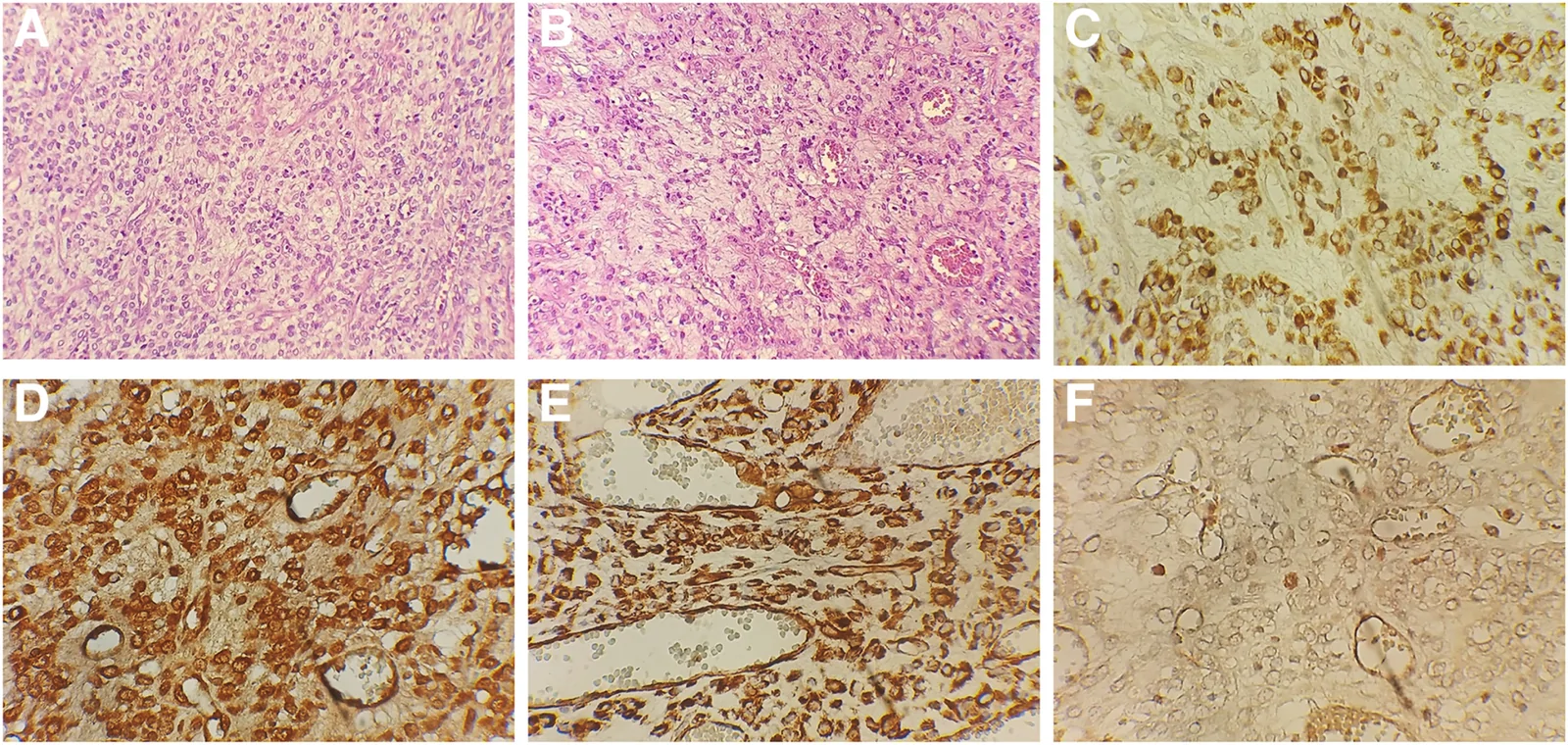
Histopathological and immunohistochemical features of the endobronchial epithelioid leiomyoma. (A) Hematoxylin and eosin-stained section showing sheets of epithelioid cells with clear-to-eosinophilic cytoplasm and minimal cytological atypia within a myxoid-like background (H&E, ×20). (B) Additional H&E section demonstrating epithelioid tumor cells with irregular open nuclei, focal stromal hyalinization, and prominent thick-walled vessels (H&E, ×20). (C) Desmin immunohistochemistry showing diffuse strong cytoplasmic positivity in the tumor cells, supporting smooth-muscle differentiation (×40). (D) Pan-cytokeratin AE1/AE3 immunohistochemistry showing focal weak cytoplasmic reactivity in a subset of tumor cells (×40). (E) Repeat vimentin immunohistochemistry demonstrating focal positivity in a small area of the tumor; the initial stain was technically noninformative because the internal control was also negative (×40). (F) Ki-67 immunohistochemistry showing a low proliferative index, with scattered nuclear labeling in <5% of tumor cells (×40). H&E = hematoxylin and eosin.

Gross pathological examination demonstrated a combined tumor mass measuring 16 × 12.6 × 5.8 cm and an endobronchial polypoid component measuring 8 × 2 × 1.5 cm. Four lymph nodes were submitted, the largest measuring 0.9 cm. Histologically, the tumor was composed predominantly of sheets and nests of epithelioid cells with clear-to-eosinophilic cytoplasm and irregular open nuclei within a myxoid-like and focally hyalinized stroma. Thick-walled vessels were also present (Fig. 6A and B). Cytological atypia was minimal, and mitotic activity was <2 mitoses/10 high-power fields. Areas of coagulative necrosis were identified. No vascular or lymphatic invasion was present.

Immunohistochemical examination demonstrated diffuse strong cytoplasmic desmin positivity (Fig. 6C) and focal weak cytokeratin reactivity in a subset of tumor cells (Fig. 6D). Smooth muscle actin (SMA) was negative despite appropriate staining of the internal positive control. The initial vimentin stain was technically noninformative because the internal control was also negative; repeat staining demonstrated focal positivity in a small area of the tumor (Fig. 6E). CD34, S100, Melan-A, CD68, chromogranin, and synaptophysin were negative. The Ki-67 labeling index was below 5%, with only scattered nuclear staining (Fig. 6F). The bronchial, pleural, and circumferential resection margins were free of tumor, and all 4 examined hilar and mediastinal lymph nodes were negative for tumor involvement. The combined morphological and immunohistochemical findings supported the diagnosis of an endobronchial epithelioid leiomyoma, historically termed leiomyoblastoma.

**Figure 7.**
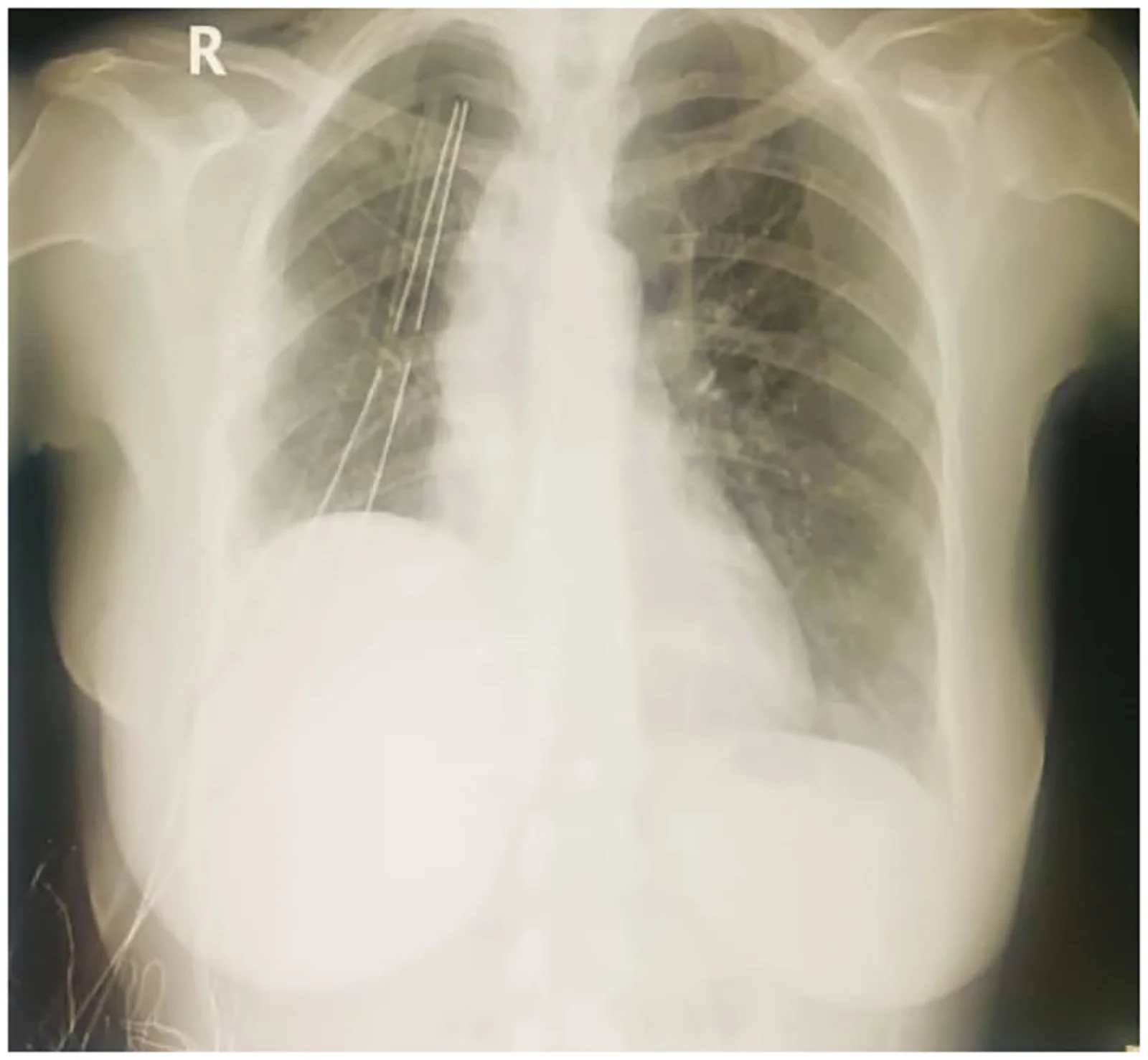
Immediate postoperative chest radiograph. Portable chest radiograph showing satisfactory re-expansion of the remaining right lung, with expected post-bilobectomy volume loss and mild rightward mediastinal shift. The chest drain is appropriately positioned, without significant pneumothorax or pleural effusion.

The early postoperative course was uneventful. The patient remained hemodynamically stable and afebrile, with adequate oxygenation on low-flow supplemental oxygen. Pain was adequately controlled, and pharmacological deep-vein thrombosis prophylaxis was initiated because of her previous postoperative thrombosis. Chest physiotherapy, incentive spirometry, and early ambulation were also commenced. Immediate postoperative chest radiography demonstrated satisfactory re-expansion of the remaining right lung, with expected post-bilobectomy volume loss and mild rightward mediastinal shift. The chest drain was appropriately positioned, without significant pneumothorax or pleural effusion (Fig. 7).

At the 2-month postoperative follow-up, the patient was clinically well and free of respiratory symptoms, and chest radiography showed a satisfactory postoperative appearance. Surveillance chest CT, repeat bronchoscopy, and gynecological evaluation were recommended, particularly because the histopathological findings from her previous uterine myomectomy were unavailable. However, she did not complete the proposed investigations. At the most recent clinical contact, approximately 13 months after surgery, she remained free of respiratory symptoms. The clinical timeline is presented in Table 1.

**Table 1:** Clinical timeline.

Date/time point	Clinical event	Key findings and management
February 2025	Symptom onset	Progressive exertional dyspnea developed over several months, reaching mMRC grade III, with persistent dry cough. Bronchodilator and corticosteroid therapy provided only partial relief.
June 4, 2025	Chest radiography	Complete opacification of the right hemithorax with ipsilateral tracheal deviation, consistent with complete right-lung atelectasis.
June 5, 2025	Non-contrast chest CT	Total right-lung collapse with marked rightward mediastinal shift, elevation of the right hemidiaphragm, and minimal right pleural effusion.
June 11, 2025	Flexible bronchoscopy	A smooth, white, cyst-like lesion completely occluded the right main bronchus. Biopsy was deferred because an endobronchial hydatid cyst and a vascular tumor such as carcinoid were considered, with concern for airway flooding, anaphylaxis, or major bleeding.
Preoperative period	Laboratory evaluation and antimicrobial therapy	Leukocyte count was 16,000/mm^3^ with 85% neutrophils. Sputum culture grew Staphylococcus aureus susceptible to linezolid. A 1-wk course improved the leukocyte count, and repeat preoperative sputum culture was sterile.
June 17, 2025	Definitive surgery	Right posterolateral thoracotomy and adhesiolysis were performed. The tumor arose from the right upper-lobe bronchus and obstructed the right main bronchus. Right upper and middle bilobectomy, extraction of the endobronchial polyp, and hilar and mediastinal lymph-node dissection were completed; the preserved right lower lobe re-expanded satisfactorily.
June 17–July 23, 2025	Histopathology and immunohistochemistry	The combined mass measured 16 × 12.6 × 5.8 cm, with an 8 × 2 × 1.5 cm endobronchial polyp. Histology showed an epithelioid smooth-muscle neoplasm with minimal atypia, <2 mitoses/10 HPFs, focal coagulative necrosis, and no lymphovascular invasion. Margins and 4 lymph nodes were negative. Desmin was diffuse; cytokeratin and repeat vimentin were focal; SMA and the remaining tested markers were negative; Ki-67 was <5%. Findings supported endobronchial epithelioid leiomyoma (leiomyoblastoma).
Early postoperative period	Recovery and chest radiography	Recovery was uncomplicated. Postoperative radiography showed satisfactory re-expansion of the remaining right lung, expected post-bilobectomy volume loss, and no significant pneumothorax or pleural effusion.
2–13 mo after surgery	Follow-up	At 2 mo, the patient was clinically well and chest radiography was reported as normal. At the most recent contact, approximately 13 mo after surgery, she remained free of respiratory symptoms. Recommended surveillance chest CT, repeat bronchoscopy, and gynecological evaluation were not completed.

Exact dates are shown where documented; relative time points are used when the precise date was unavailable.

CT = computed tomography, HPF = high-power field, mMRC = modified Medical Research Council, SMA = smooth muscle actin.

## 3. Discussion

The term *leiomyoblastoma* was historically used to describe an unusual gastric smooth-muscle neoplasm that resembled a leiomyoma grossly but displayed predominantly epithelioid histological features. The designation itself did not imply malignancy, and benign and malignant forms were subsequently described under several overlapping terms, including *epithelioid leiomyoma* and *bizarre leiomyoma*.^[5]^ Because *leiomyoblastoma* may misleadingly suggest an immature or primitive neoplasm, later authors favored the more descriptive term *epithelioid leiomyoma*.^[7]^ Accordingly, we use *endobronchial epithelioid leiomyoma (leiomyoblastoma*) consistently in this report. Although similar epithelioid smooth-muscle tumors have been described in the stomach, uterus, tongue, round ligament, vulva, urethra, and other soft-tissue sites, their morphological and ultrastructural characteristics may vary by anatomical location.^[4,7–12]^ Endobronchial involvement remains exceptionally rare, and only 1 earlier publication specifically designated as endobronchial leiomyoblastoma was identified in the available literature.^[6]^

Benign endobronchial tumors are uncommon and may remain undetected until progressive obstruction produces substantial distal pulmonary damage. Their symptoms and radiological findings are nonspecific, and atelectasis, post-obstructive infection, bronchiectasis, or mediastinal shift may mimic malignant airway disease.^[1–3]^ Bronchial leiomyoma may similarly appear as a smooth or lobulated central lesion and cannot be reliably distinguished from carcinoid tumor or other endobronchial neoplasms by imaging alone.^[1]^ The severity of the presentation in the current case was therefore attributable primarily to the tumor’s critical location and prolonged complete bronchial obstruction rather than to histological aggressiveness.

The pathological diagnosis was supported by the predominance of round-to-polygonal epithelioid cells arranged in sheets and nests, with clear-to-eosinophilic cytoplasm, minimal atypia, low mitotic activity, and areas of stromal hyalinization. These features correspond to the reported morphological spectrum of epithelioid smooth-muscle tumors.^[4,7,8,10–13]^ In contrast, conventional bronchial leiomyoma is typically composed of intersecting fascicles of bland spindle cells with cigar-shaped nuclei and commonly expresses desmin, SMA, and h-caldesmon.^[1]^ Thus, conventional bronchial leiomyoma and the present tumor belong to the same broad smooth-muscle lineage but are distinguished by their predominantly spindle and epithelioid morphologies, respectively. Ultrastructural findings such as cytolysosomes, glycogen aggregates, and smooth-muscle myofilaments have been described in uterine clear-cell epithelioid leiomyomas; however, electron microscopy was not performed in our case, and these site-specific observations should not be directly attributed to this tumor.^[7]^

The immunohistochemical findings should be interpreted in the context of the recognized phenotypic variability of epithelioid smooth-muscle tumors. Reported epithelioid smooth-muscle tumors commonly express smooth-muscle markers such as desmin and SMA, with vimentin positivity documented in some cases.^[8,10–12]^ Nevertheless, immunophenotypic variability has been reported,^[5,8,13]^ and focal cytokeratin expression may occur in epithelioid smooth-muscle neoplasms.^[8,13]^ Thus, diffuse desmin positivity with focal weak cytokeratin reactivity and negative SMA staining does not, in isolation, contradict smooth-muscle differentiation. The preserved SMA internal control supported a true negative result, while repeat vimentin staining demonstrated focal tumor-cell positivity after the initial stain was technically noninformative. The final interpretation therefore relied on the combined morphology, diffuse desmin expression, focal vimentin positivity, low proliferative activity, and exclusion of the principal morphological mimics rather than on any single immunohistochemical marker.

Focal coagulative necrosis was the most concerning pathological feature. Criteria developed for uterine epithelioid smooth-muscle tumors generally define benign epithelioid leiomyoma by the absence of significant atypia, increased mitotic activity, and tumor-cell necrosis.^[13,14]^ However, these criteria have not been validated for primary bronchial epithelioid smooth-muscle tumors and cannot be directly applied to this exceptionally rare location. In this case, <2 mitoses/10 high-power fields, minimal cytological atypia, a Ki-67 index below 5%, absence of lymphovascular invasion, negative lymph nodes, and complete tumor-free margins collectively favored an indolent epithelioid smooth-muscle neoplasm. Although reassuring, these findings do not guarantee an entirely benign long-term course. Historical reports have similarly emphasized that the behavior of leiomyoblastoma may not always be predicted from a single morphological feature and that continued follow-up is important.^[5]^

The principal differential diagnoses included conventional bronchial leiomyoma, carcinoid tumor, glomus tumor, inflammatory myofibroblastic tumor, and perivascular epithelioid cell tumor (PEComa). Conventional bronchial leiomyoma was distinguished by the absence of the characteristic spindle-cell fascicular architecture described above.^[1]^ Carcinoid tumor was excluded by the absence of neuroendocrine morphology and negative chromogranin and synaptophysin staining.^[1]^ Glomus tumor was strongly disfavored by negative SMA staining and diffuse desmin positivity. PEComa was disfavored by the negative Melan-A and SMA results and the overall smooth-muscle morphology; PEComas frequently express melanocytic markers, particularly Melan-A and HMB45, whereas conventional epithelioid smooth-muscle tumors usually lack Melan-A expression.^[13,14]^ Because HMB45 was not performed, complete immunohistochemical exclusion of PEComa cannot be claimed. Inflammatory myofibroblastic tumor was not supported by the absence of a prominent inflammatory myofibroblastic proliferation, infiltrative growth pattern, or characteristic stromal changes. ALK testing may assist in challenging cases but was not performed.^[13]^

Bronchoscopy with tissue sampling is usually important for establishing a preoperative diagnosis and selecting the least extensive definitive treatment. A confirmed diagnosis of bronchial leiomyoma may permit bronchoscopic excision, bronchotomy, or bronchoplastic resection with preservation of functional lung tissue.^[1,3]^ In this patient, however, the smooth, cyst-like appearance raised concern for an endobronchial hydatid cyst. Biopsy of a suspected hydatid lesion could have caused rupture, airway contamination, and anaphylaxis. A vascular lesion, particularly a carcinoid tumor, also remained possible and introduced a risk of major endobronchial hemorrhage. This concern was clinically important because the right main bronchus was completely obstructed, and effective ventilation depended on the contralateral lung. Severe bleeding during bronchoscopic assessment of endobronchial tumors has been reported and may necessitate urgent surgical intervention.^[15]^ Deferring biopsy was therefore a case-specific safety decision rather than a departure from the general value of preoperative histological diagnosis.

The treatment of benign endobronchial tumors should achieve complete removal while preserving as much functional lung parenchyma as anatomy permits. Bronchoscopic resection is appropriate for selected small, purely intraluminal lesions, whereas bronchotomy, sleeve resection, lobectomy, or bilobectomy may be required according to tumor size, attachment, extrabronchial extension, and the condition of the distal lung.^[1–3,15]^ In the current case, sleeve or bronchoplastic resection would not have preserved useful upper-lobe tissue because that lobe had been extensively destroyed and replaced by tumor. The incompletely developed fissure also made the upper and middle lobes an integrated anatomical unit. Upper and middle bilobectomy therefore provided complete resection while preserving the uninvolved bronchus intermedius and functional right lower lobe. This approach reflects the principle that parenchymal preservation should be pursued when meaningful but should not compromise complete excision or retain irreversibly damaged lungs.

The history of uterine myomectomy introduces an additional diagnostic limitation. Benign metastasizing leiomyoma can produce histologically bland pulmonary smooth-muscle lesions in women with previous uterine leiomyomas, sometimes many years after the original gynecological operation.^[13,14]^ Its diagnosis requires exclusion of a uterine or extrauterine leiomyosarcoma and ideally confirmation that the original uterine lesion was thoroughly sampled and histologically benign.^[14]^ Pulmonary benign metastasizing leiomyoma more commonly presents with multiple bilateral parenchymal nodules rather than a solitary polypoid lesion arising within a main bronchus.^[13,14]^ The isolated endobronchial growth pattern in this patient therefore favored a bronchial origin. However, because the original uterine histopathological material was unavailable, a relationship to the previous uterine smooth-muscle tumor cannot be excluded with certainty. For this reason, the designation of the lesion as unequivocally *primary* should be used cautiously.

This report is limited by its single-case design, the absence of previous uterine pathological material, and the incomplete ancillary panel for some differential diagnoses, including the lack of HMB45 and ALK testing. Follow-up is also predominantly clinical. A satisfactory radiograph was available at 2 months, and the patient remained free of respiratory symptoms approximately 13 months after surgery; however, the recommended surveillance CT and bronchoscopy were not completed. Clinical stability alone cannot exclude a small residual or recurrent endobronchial lesion. Continued clinical, radiological, and bronchoscopic surveillance is therefore warranted, particularly because of the tumor’s exceptional rarity, focal coagulative necrosis, uncertain long-term biological behavior, and the unresolved relationship to the previous uterine lesion.^[1,5]^

## 4. Conclusion

Endobronchial epithelioid leiomyoma (leiomyoblastoma) is an exceptionally rare cause of complete unilateral lung collapse. In this patient, extensive destruction of the right upper lobe and unfavorable fissural anatomy precluded meaningful parenchymal-sparing resection, necessitating right upper and middle bilobectomy while preserving the functional lower lobe. The predominantly epithelioid morphology, diffuse desmin expression, low mitotic activity, low Ki-67 index, negative lymph nodes, and clear resection margins supported an indolent smooth-muscle neoplasm. However, focal coagulative necrosis, unavailable pathology from the previous uterine myomectomy, and incomplete postoperative surveillance warrant diagnostic caution and continued long-term clinical, radiological, and bronchoscopic follow-up. Complete resection remains the principal treatment, with bronchoscopic or bronchoplastic parenchymal-sparing approaches preferred when anatomically feasible.

## Author contributions

**Data curation:** Bassel Ibrahim.

**Formal analysis:** Bassel Ibrahim.

**Investigation:** Mais Salleh.

**Methodology:** Bassel Ibrahim.

**Supervision:** Youness Souleiman.

**Writing – original draft:** Raneem Ahmad, Youssef Abbas, Mohammad Alaa Aldakak.

**Writing – review & editing:** Raneem Ahmad, Youssef Abbas, Mohammad Alaa Aldakak.
